# Ectopic Ciliated Cyst in the Mandible Secondary to Genioplasty and Lefort after Two Years: A Case Report and Literature Review

**Published:** 2016-09

**Authors:** Safoura Seifi, Shabnam Sohanian, Oveis Khakbaz, Farida Abesi, Fatemeh Aliakbarpour, Arezoo Rayani

**Affiliations:** 1 *Oral Health Research Center, Babol University of Medical Sciences, Babol, Iran. *; 2 *Department of Oral and Maxillofacial Pathology Specialized, Babol University of Medical Sciences, Babol, Iran. *; 3 *Department of Oral and Maxillofacial Surgery, Faculty of Dentistry, Babol University of Medical Sciences, Babol, Iran. *; 4 *Department of Oral and Maxillofacial Radiology, Faculty of Dentistry, Babol University of Medical Sciences, Babol, Iran. *

**Keywords:** Ciliated cyst, Mandible, Genioplasty

## Abstract

**Introduction:**

The ectopic ciliated cyst is a rare non-odontogenic cyst which occurs as a delayed complication after maxillary sinus radical surgery; this lesion emerges due to the destruction of the sinus mucosa during the surgery and entrapment of the respiratory epithelium in the region. This lesion has been observed in very rare cases following genioplasty and bimaxillary orthognathic surgery.

**Case Report:**

We reported a case of the ectopic ciliated cyst in in the mandible of a 37-year-old Iranian woman following genioplasty and Lefort 1 surgery after 2 years. Its treatment was enucleation.

**Conclusion:**

Long-term follow-up after cosmetic surgery of both jaws is recommended due to the probability of this cyst.

## Introduction

The ectopic ciliated cyst, which is also known as the post operative maxillary cyst and implantation cyst, comprises about 1.5 % of total oral cysts and is often developed as a delayed complication (6 to 49 years) after maxillary sinus surgery as a result of respiratory epithelium getting trapped in the surgical site (1-3). Most often, it occurs at the posterior maxilla and in the mandible in rare cases. The most common operations related to the cyst are Lefort I- III, medial osteotomy of the face, maxillary sinus surgery, traumatic dental extraction, maxillary fractures, and genioplasty cosmetic surgery (4). The age at which it occurs is the second and third decade of life and it is observed more in Asian and Japanese races. This cyst is asymptomatic in the beginning, but symptoms such as swelling and cheek pain or mucobuccal fold may be seen later due to its invasive nature (5). This cyst is usually specified as a radiolucent lesion with definite borders on radiography (6). Histopathologicaly, the cyst has ciliated cubic or cylindrical epithelium in 66% of the cases, and a stratified squamous epithelium or a mixed epithelium in some cases. The odds of relapse are low; however, if the cyst is not completely removed, it may relapse in about 20% of the cases (7). 

This is the 11th report, in English papers, of an ectopic ciliated cyst of the mandible following genioplasty and Lefort I after 2 years of surgery in the upper and lower mandible. 

## Case Report

**Fig 1:**
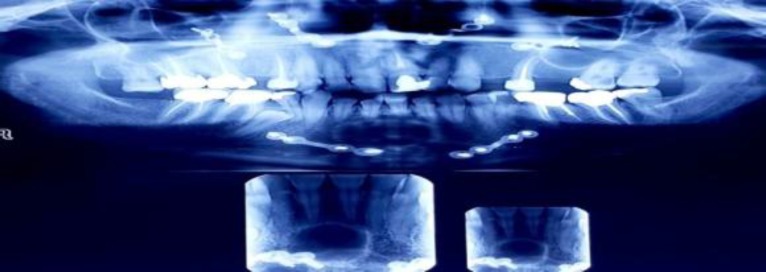
Panoramic view, unilocular well defined radiolucency in anterior of mandible after 2 years genioplasty

The patient was a 37-year-old Iranian woman who had visited the surgery unit of the dentistry department due to mild inflammation in the anterior mandible area. She did not mention any systematic diseases or the use of special drugs but reported Menu Max and genioplasty (advancement of maxilla) and Lefort 1 surgery for cosmetic reasons 2 years ago (Fig. 1). 

**Fig 2:**
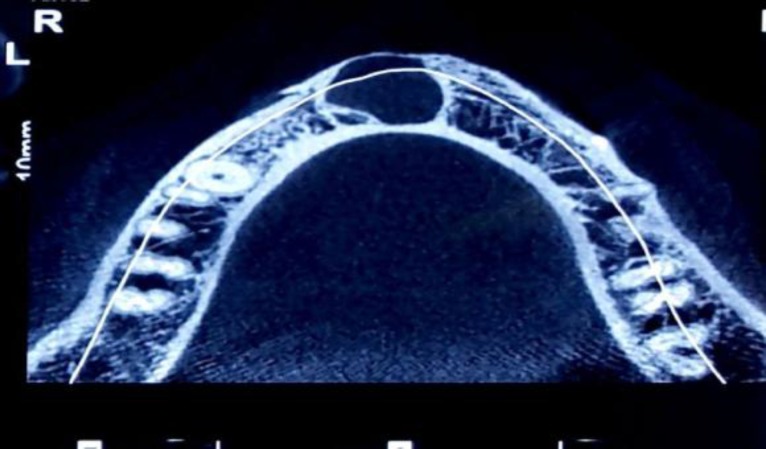
Occlusal view, mild expansion of anterior of mandible

Upon the panoramic radiography view, a unilocular radiolucency with definite borders was seen in the anterior mandible area which was developed from the second right tooth to the second left tooth (Fig.2).

**Fig 3:**
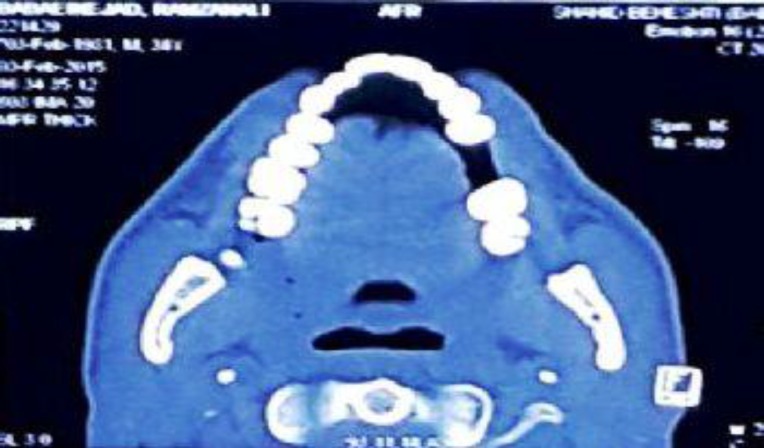
CT scan, unilocular radiolucency in anterior of mandible

Upon CT scan, the lesion had led to the perforation of the mandibular buccal bone in the anterior area (Fig. 3).

Considering the clinical signs and symptoms and radiographic images, differential diagnoses included traumatic bone cyst, glandular odontogenic cyst, odontogenic keratocyst, and central giant cell granuloma. The sample was sent to the pathology laboratory in formalin 10%.

Considering the clinical signs and symptoms and radiographic images, differential diagnoses included traumatic bone cyst, glandular odontogenic cyst, odontogenic keratocyst, and central giant cell granuloma. The sample was sent to the pathology laboratory in formalin 10%.

Upon macroscopic investigation, a piece of plate shaped soft gray cream tissue with elastic consistency measuring 0.3 × 0.5 × 0.7 cm in size was seen. Cross-sectional cutting of the cyst showed that the cross section was non-uniform in section. A cystic section with a non-uniform thickness of at least 1 mm was observed.

**Fig 4:**
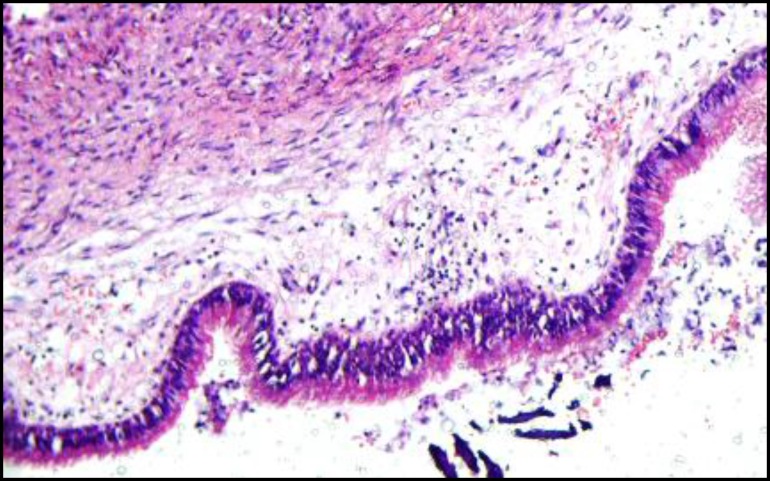
a cystic stratified structure with a ciliated respiratory epithelium. Hematoxylin-eosin staining(x10)

**Fig 5:**
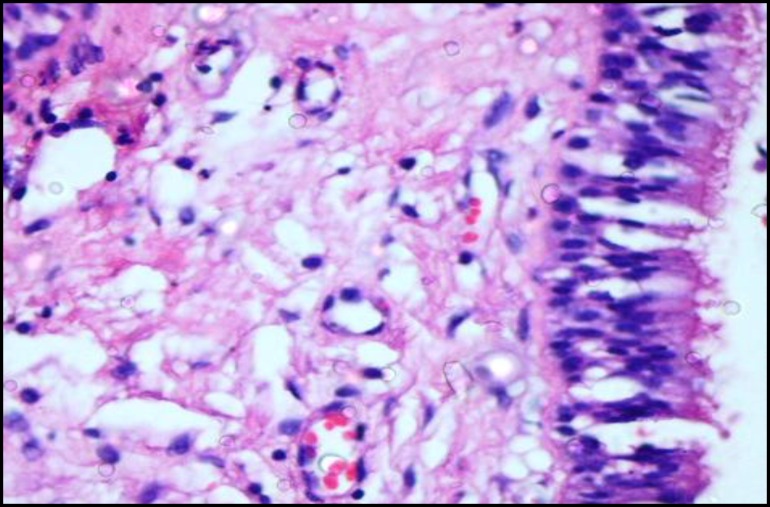
Histopathologic view of surgical ciliated cyst (x40).

Upon microscopic investigation, a cystic stratified structure with a ciliated respiratory epithelium or sometimes stratified squamous epithelium with mucous cells lacking rete peg Ectopic Ciliated Cyst in the Mandible were observed. The underlying connective tissue was fibrous and collagenized and had delicate blood vessels and few inflammatory cells. Based on pathological findings and location of the lesion, a diagnosis of ectopic ciliated cyst was made (Figs 4,5).

## Discussion

The ectopic ciliated cyst is a non-odontogenic and common cyst in the maxilla, which develops after sinus surgery or traumatic dental extraction. In very rare cases, it may occur in the mandible (8). 10 cases have been reported in English literature (1). 

In our case, the cyst was detected in a 37-year-old woman that presented with mild swelling without fistula formation. Review of the literature showed that of the 11 cases that had the cyst in the mandible, 5 were female and 6 were male with an age of onset of 24-72 years (1). This cyst is common in East Asia, especially Japan, but it is very rare in European countries (8). Its real etiology is unknown in the mandible. It is believed that it may be created in two jaws after orthogenetic or Lefort surgery (5). In this report, the patient had undergone genioplasty and Lefort surgery and perhaps after using one blade in the surgery of both jaws or a graft transfer, some pieces of the maxillary sinus epithelium (respiratory epithelium) fell into the mandible leading to the cyst formation.

In 2014, Li et al reported two cases of ciliated surgery cyst in the mandible after rhinoplasty, genioplasty*,* and Lefort surgery in men aged 42-72 years. The second man had a history of surgery 56 years ago and the first man underwent surgery 8 years ago. Some symptoms such as painless swelling and numbness of the chin were mentioned as the symptoms of this cyst (1). In studies by Nastria, Anastassor, Imholte, Ragsdale, Li, this cyst was observed in the anterior mandible, similarly to our patient (1,9-12). However, in studies by Bourgeose Houtlas and Lazar) 5,13), it was reported in the posterior mandible. In most reported cases, the main symptoms were swelling and sometimes pain. Bourgeose et al discussed the cyst as an asymptomatic lesion while Li described paresthesia as one of its symptoms (5). In our case, perforation of the buccal cortex was observed similarly to the case reported by Bougeois et al (5). In all the cases with lesions in the mandible, there is a positive history of genioplasty, rhinoplasty, Lefort I, or orthognathic surgery 4-56 years before the development of the cyst (1). In our patient, there was a positive history of genioplasty and Lefort I surgery 2 years before the lesion developed. Moreover, radiography revealed an anterior mandibular radiolucent lesion with well defined borders. The differential diagnoses of the lesion included traumatic bone cyst, glandular odontogenic cyst, keratocyst, and central giant cell granuloma. The last differential diagnosis was ruled out after positive aspiration and drainage of the fluid. Upon pathological evaluation, due the presence of ciliated stratified columnar epithelium along with stratified squamous epithelium with mucosal and goblet cells, a diagnosis of surgical ciliate cyst was proposed. In keratocysts, there is parakera- tinized stratified squamous epithelium, and in glandular odontogenic cysts, there is stratified squamous epithelium upon which ciliated cylindrical columnar epithelium can be visualized. It seems that if the surgeon performs cosmetic surgery of the mandible and maxilla separately or uses clean blades separately for cosmetic surgery of the maxilla, there will be no risk of the transfer of the mucosal epithelium of the sinus to the mandible and formation of the surgical ciliated cyst.

In our patient, because the ciliated cyst developed in the mandible, the term ectopic ciliated cyst was used. Therefore, long-term follow-up of patients who undergo cosmetic or genioplasty surgery is necessary due to the probability of the formation of such cysts. Enucleation is performed in most patients who have an ectopic ciliated cyst in the mandible. Ragsdale suggested enucleation and peripheral ostectomy, and Li et al suggested abortion (1).

## Conclusion

Ectopic ciliated cyst is a rare non-odontogenic cyst in the mandible following genioplasty and orthognathic and Lefort surgery. Therefore, long-term follow-up of both jaws is recommended after cosmetic surgery.
